# The interplay of migration and cultural similarity between countries: Evidence from Facebook data on food and drink interests

**DOI:** 10.1371/journal.pone.0262947

**Published:** 2022-02-09

**Authors:** Carolina C. Vieira, Sophie Lohmann, Emilio Zagheni, Pedro O. S. Vaz de Melo, Fabrício Benevenuto, Filipe N. Ribeiro

**Affiliations:** 1 Laboratory of Digital and Computational Demography, Max Planck Institute for Demographic Research, Rostock, Germany; 2 Computer Science Department, Federal University of Minas Gerais, Belo Horizonte, Brazil; 3 Computer and Systems Department, Federal University of Ouro Preto, Ouro Preto, Brazil; University of Oklahama Norman Campus: The University of Oklahoma, UNITED STATES

## Abstract

Migration has been proposed as one of the factors that shape cultural similarities across countries. However, studying the relationship between culture and migration has been challenging, in part because culture is difficult to quantify. The traditionally used survey questionnaires have a number of drawbacks, including that they are costly and difficult to scale to a large number of countries. To complement survey data, we propose the use of passively-collected digital traces from social media. We focus on food and drink as markers of a country’s culture. We then measure similarities between countries in terms of food and drink interests using aggregated data from the Facebook Advertising Platform. Methodologically, we offer approaches to measure the similarity between countries with both symmetric and asymmetric indices. Substantively, we assess the association between migration cultural similarity between countries by comparing our measure of cultural similarity with international migration data. In most countries, larger immigrant populations are associated with more similar food and drink preferences between their country of origin and the country of destination. Our results suggest that immigrants contribute to bringing the culture of their home countries to new countries. Moreover, our study identifies considerable variability in this pattern across countries.

## Introduction

Açaí bowls are a very popular food in the United States. According to *The Daily Meal* media outlet, açaí bowls were the trendiest breakfast of 2020 (https://www.thedailymeal.com/news/trendiest-breakfast-of-2020/070920). How can we explain this when Brazil, the home country of açaí, is thousands of miles away from the US?

Despite far distances and differences between cultures, many populations share cultural preferences. Cultures are influenced by economic, political, and demographic changes, with migration being one of the main drivers of cultural changes across countries [1]. However, it has been difficult to measure the association between culture and international migration because cultural data that can be compared across multiple countries are scarce.

In this paper we propose a scalable methodology to measure cultural similarity between countries by using data from social media. Our proposed measure of cultural similarity also considers other characteristics such as international data availability, time, cost, and asymmetry (e.g. country *A* may adopt features of country *B*’s culture, but country *B* may not adopt features of country *A*’s culture), which have hampered previous efforts to study cultural similarity. Moreover, because migration plays a crucial role in shaping cultural similarity between countries, as an additional contribution we show how our approach can be used to quantify the association between cultural similarity and international migration across countries.

The relationship between migration and culture is bidirectional: cultural fit is one of the most important factors that people consider before moving to a new destination country [2] and migrants transmit cultural elements from their origin country to their destination country and back [3]. However, most of the studies analyzing cultural changes due to migration are restricted to one or a few countries or, when considering an international perspective of the effect of migration on cultural dynamics, the cultural distance measures used are by construction symmetric [4]. Since migration is neither homogeneous across countries nor symmetric, we develop non-symmetric measures of cultural similarity across a large number of countries to more accurately represent processes of international cultural exchange.

Moreover, other factors complicate the comparison between cultural similarity and migration. First, to measure cultural similarity between countries we need a measure of cultural similarity or cultural distance [5]. Cultural distance measures refer to operational parameters that can be used as proxies for cultural dimensions and allow estimating scores to gauge the extent to which countries differ culturally [6]. The cultural dimensions used to measure culture can vary depending on the focus of the research [7].

Operationally, culture has been traditionally measured via sampling surveys [8] in which the survey responses are used to characterize cultural aspects of a country. Several studies, such as Schwartz’s value survey [9], the World Values Survey [10], and Hofstede’s cultural characteristics (https://www.hofstede-insights.com/models/national-culture/) [11] try to identify cultural dimensions related to values on which countries tend to differ. Moreover, some methods derived from surveys propose evaluating the relative distance between countries [12–15].

However, the study of culture can also focus on aspects of our daily life by considering cultural objects such as the clothes we wear, the music we listen to, and the food we eat [8, 16]. Food studies are an established interdisciplinary field that recognizes the centrality of food for cultural practices and cultural identity [17, 18]. Several studies have explored how food communicates our culture and the mechanisms by which we relate food to our cultural identities, whereas others revealed that people and groups can be discriminated against on the basis of their food and cultural habits [19–21]. Similarly, Almerico [20] presented an interdisciplinary study that documents the intricate relationships between food, culture, and society from a sociological perspective. Typical food from a country, for example, can be used to approximate cultural distance by characterizing the preferences for similar food in other countries. Cantarero *et al.* [22] show that cultural identity greatly influences food choices by performing a qualitative and quantitative analysis in the Comunidad Autónoma de Aragón, Spain. They found that people prefer to consume foods that are symbolically associated with their own culture to reinforce their sense of belonging. In summary, these previous studies are important to create a strong connection between the culture of a country and food, our cultural marker to measure cultural similarity between countries.

Such studies have typically been based on surveys, but surveys have important limitations. For example, in addition to measurement error [23], results may suffer from various biases [24] like social desirability bias, question order bias, and acquiescence bias. Furthermore, surveys are costly and require a long time to run. To overcome part of these limitations, we propose an approach that relies on passively-collected data from social media.

Social media platforms provide complementary tools that can be used to measure cultural preferences and compare regions via passively-collected data [25]. As one of the first studies to address this question by using online data sources, Silva *et al.* [26] identified cultural boundaries and similarities across populations by clustering them based on the analysis of food and drink habits. However, their analyses of culinary habits around the world were limited to Foursquare check-ins, which considered only 101 interests and, consequently, underestimate the true breadth of users’ interests and of cultural variation. Moreover, Foursquare is not widely used and demographically biased (e.g., https://www.statista.com/statistics/814726/share-of-us-internet-users-who-use-foursquare-by-age/), especially because Silva *et al.* only gathered data from Foursquare users who were also Twitter users and decided to cross-post their Foursquare check-ins to Twitter.

Other studies have used data provided by the Facebook Advertising Platform. With more than 2.7 billion worldwide users (https://www.facebook.com/iq/insights-to-go/2740m-facebook-monthly-active-users-were-2740m-as-of-september-30/), Facebook captures a larger and more diverse population than other social media. A growing literature has successfully used Facebook Ads data to study many different topics such as migration [27–29], the relationships between immigrant communities [30], and cultural assimilation between migrants [31]. We therefore see high potential in applying Facebook Ads data to the study of cultural similarity regarding international food and drink preferences.

In a first study in this area, Vieira *et al.* [32] examined the similarity between selected countries and Brazil based on their population’s interests in typical Brazilian food. However, the results were limited to foods listed on Wikipedia. This limits the potential list of food and, importantly, some countries do not have a Wikipedia page dedicated to list their typical food. Here, we propose a more scalable, data-driven methodology to identify the most popular foods and drinks in each country from a list containing *more than 200,000 interests* on Facebook Ads [33].

More recently, Obradovich *et al.* [34] examined cross-national cultural differences across nearly 60,000 topic dimensions from Facebook Ads. They also validated their work by comparing the cultural distance calculated from their measurement with traditional survey-based measures. However, the authors used different types of features from Facebook Ads, which is a ‘black-box’ model, to represent countries in terms of culture. In contrast, we propose a methodology to select the most important cultural attributes, in this case, related to food and drink for each country from a data set containing more than 200,000 interests of Facebook users. In this case, we choose to compare countries in terms of fewer, but explicitly known attributes selected from a very large data set through a data-driven approach. In other words, interests that are not relevant to any of the countries are disregarded in order to reduce feature sparsity. Finally, we additionally present the first work exploring a non-symmetric measure of cultural similarity and further assess the association between migration and food- and drink-based cultural similarity across countries.

We expand the methodology proposed by Vieira et al. (2020) [32] to (i) propose a scalable approach based on data from the Facebook Advertising Platform (Facebook Ads) to obtain proxies for culture to measure cultural similarity between countries; (ii) develop measures of cultural similarity to compare countries according to their population’s interests revealed by Facebook; (iii) assess to which extent cultural similarity between countries is associated with migration.

Culture encompasses many aspects, including preferences for music, art, and food. Although in this paper we focus on food and drink as markers of culture [16], we offer a methodological contribution that can be applied to other cultural markers. Moreover, we provide a substantive contribution of results for the case of food and drink. Finally, we hypothesize a link between migration and food and drink preferences revealed by social media. We provide initial results that lead to further avenues for research in this area at the intersection of social computing, demography, and sociology.

## Materials and methods

In this section, we present the data and methodology proposed in this work. Data collection was performed in compliance with the terms of services of the websites from which data was collected. All data used in this study are openly available and the data sources are specified in each subsection, including the data from Facebook, openly available through Facebook’s Marketing Application Programming Interface (API) (https://developers.facebook.com/docs/marketing-apis). However, due to legal requirements regarding the publication of the raw data collected from the Facebook Advertising Platform data, the minimal data underlying the results of this study are available for academic purposes upon request. All the measures derived from the Facebook Ads data and the code to analyze the data sets and generate the figures are available here: https://github.com/carolcoimbra/cultural-similarity-fb

### Data

Because food is recognized as a central cultural marker [16–18] and there is a great variety of popular local food and drink in each country, we decided to use the data categorized by Facebook Ads as related to *Food and drink* from the data set collected via *snowball* [33] containing most of the interests available on Facebook Ads in 2019.

Following Silva *et al.*’s [26] prior work in this area, we selected a subset of 16 countries (Argentina, Australia, Brazil, Chile, Great Britain, France, Indonesia, Japan, South Korea, Malaysia, Mexico, Russia, Singapore, Spain, Turkey, and the United States). The analysis is limited to the subset of countries we consider, but following the methodology proposed, it is possible to extend the analysis to include any other country with a Facebook audience.

Regarding privacy, our work uses only aggregated data and we do not gather nor link any personal information to any particular user. The methodology adopted to gather the data as well as the audience of each food and drink over Facebook users from each country through the Facebook Advertising Platform is described in the following section, where we also present other data sets that we use to assess our proposed methodology.

#### Facebook Ads data

The Facebook Marketing API allows marketers and researchers to obtain an estimate of the number of monthly active users for a proposed advertisement that matches given input criteria [35]. For that, the platform provides a list of demographic attributes, such as age, gender, home location, and interests that can be customized by the advertiser as the input query. Thus, after specifying the target audience, and before the ad is launched, advertisers are provided with the size of the audience that matches the target specifications.

Attributes like age, gender, and location are explicitly declared by the users in their profiles, whereas interests can be either declared by the user or inferred by Facebook based on user activities such as posting or interacting with contents (e.g., liking content, sharing content, or updating one’s status). Facebook users generate traces of their preferences in multiple domains. The interests are categorized in one of the following categories: *People*, *Education*, *News and entertainment*, *Travel, places and events*, *Food and drink*, *Business and industry*, *Technology*, *Hobbies and activities*, *Sports and outdoors*, *Lifestyle and culture*, *Shopping and fashion*, *Fitness and wellness*, *Family and relationships* [28] such as music [31] and food [32].

Once not all the 258,366 interests collected via *snowball* [33] are related to food and drink, we selected 9,309 categorized by Facebook Ads as referring to *Food and drink* interests. However, only 996 interests related to *Food and drink* have an audience higher than 1,000 in each one of the countries considered. Note that 1,000 is the minimum value given by the Facebook Ads API, meaning that the true value can be anywhere between 0 and 1,000. We adopted this cutoff to reduce data sparsity and include only interests that can be meaningfully compared across all countries in our sample. Because the Facebook audience in each country is in the order of millions, this threshold is further low enough that popular interests relevant to our analyses would not be erroneously excluded. Moreover, from those 996 interests, many included restaurants (e.g., Burger King), kitchen utensils (e.g., spoon), and specific brands (e.g., Pepsi). To consider only interests which represent the names of food, drinks, ingredients, or dishes, we manually validated the data set by removing other interests such as brand names. At the end of this process, 728 interests were considered in the following analysis. According to Facebook’s Terms of Service, the raw data collected from the Facebook Advertising Platform cannot be shared publicly. Instead, all the measures derived from the Facebook Ads data and the code to analyze the data sets and generate the figures are available here: https://github.com/carolcoimbra/cultural-similarity-fb. Moreover, for reproducibility purposes, we can share the raw data upon request.

The Facebook Ads Platform does not include detailed information on how interests are estimated, and the population of Facebook users is known to be biased concerning gender, age, and other socio-demographic characteristics [36, 37]. However, many studies have already shown that this platform can be used as a good proxy to study population’s characteristics [38]. Also, previous studies showed that the subset of interests used in our work is representative [33]. Regarding privacy, our work uses only aggregated data and we do not gather nor link any personal information to any particular user. We complied with the terms of service of Facebook’s Marketing API (https://developers.facebook.com/policy/#marketingapi). In particular, the data collected is anonymous (12. j.) and we did not build or augment any user profiles (5. b. ii.). Moreover, we did not perform, or facilitate or support others in performing, any of the following prohibited practices (3. a.) listed on the new Facebook Platform Terms the new Developer Policies effective since August 31, 2020.

#### Survey data

Because we are interested in measuring cultural similarity between countries by using Facebook Ads data, it is important to compare our results with previous works that try to address similar questions. Inglehart and Welzel proposed a cultural map of the world based on one of the most prominent surveys, the World Values Surveys data. The data from the Inglehart-Welzel cultural map using the wave 7 (2017-2020) of the WVS can be downloaded here: https://www.worldvaluessurvey.org/WVSEventsShow.jsp?ID=428 [39].

The World Values Surveys data set looks at several cultural dimensions such as religion, politics, economics, and lifestyle. However, Inglehart and Welzel assert that there are two major dimensions of cross-cultural variation in the world: Traditional values (which emphasize the importance of religion, parent-child ties, deference to authority, and traditional family values) versus Secular-rational values (represented by societies which place less emphasis on religion, traditional family values and authority); and Survival values (emphasis on economic and physical security) versus Self-expression values (high priority to environmental protection, equality, and rising demands for participation in decision-making in economic and political life). Based on these two dimensions, they proposed an international cultural map where the location of each country is given by the scores on these two dimensions. Fig 5 shows the reproduction of the World Value Survey cultural map 2020 considering the selected countries.

Even if not focused on food and drink, these data can be used for comparison purposes, providing an idea of how food- and drink-based cultural markers are correlated with the value-based cultural markers estimated by the WVS. In addition, because we are using only food and drink as cultural markers, we can measure how similar our results are in comparison with one of the most complete data sources based on surveys.

#### Migration data

We also compare our results with migration data to understand the mechanisms behind the cultural similarity expressed by the Facebook users’ interests in popular food and drink from different parts of the world. The migration data refer to the total international migrant stock in 2019 by destination and origin provided by the United Nations. The data from the United Nations regarding the international migrant stock from 2019 can be downloaded here: https://www.un.org/en/development/desa/population/migration/data/estimates2/estimates19.asp [40]. These data include estimates of how many immigrants from each of one of the 232 countries and areas of the world were living in each of these 232 countries and areas of the world in 2019. The estimates are based on official statistics on the foreign-born or the foreign population.

### Methodology

In this section, we present the methodology that we developed to measure the cultural similarity between countries by exploring Facebook audiences interested in popular food and drink across countries. First, we focus on selecting a subset of popular food and drink for each country. Then, we use those interests related to food and drink to create a representation of each country as a vector to calculate the similarity between countries.

#### Popular food and drink

In order to select the subset of popular food and drink, we collected the Facebook audience interested in each food and drink. To consider only the most popular food and drink in each country, we selected the top food and drink according to their popularity among Facebook users living in the country.

Concretely, in our context, the popularity of a food or a drink among the Facebook users living in the country is measured as the proportion of users on Facebook living in each country interested in that food or drink. Eq 1 shows how we can obtain this proportion. *audience*_*c*_(*i*) represents the number of Facebook users in country *c* interested in food or drink *i*, while *audience*(*i*) represents the total audience interested in the same food or drink on Facebook as a whole. Notice that *audience*(*i*) can also be written as a sum of the Facebook audiences interested in *i* for each country, so *audience*(*i*) = ∑_c*_ *audience*_c*_(*i*).

$$\begin{array}{c} audience_{i-norm}=\frac{audience_{c}\left(i\right)}{audience(i)} \end{array}$$ (1)

**Fig 1:**
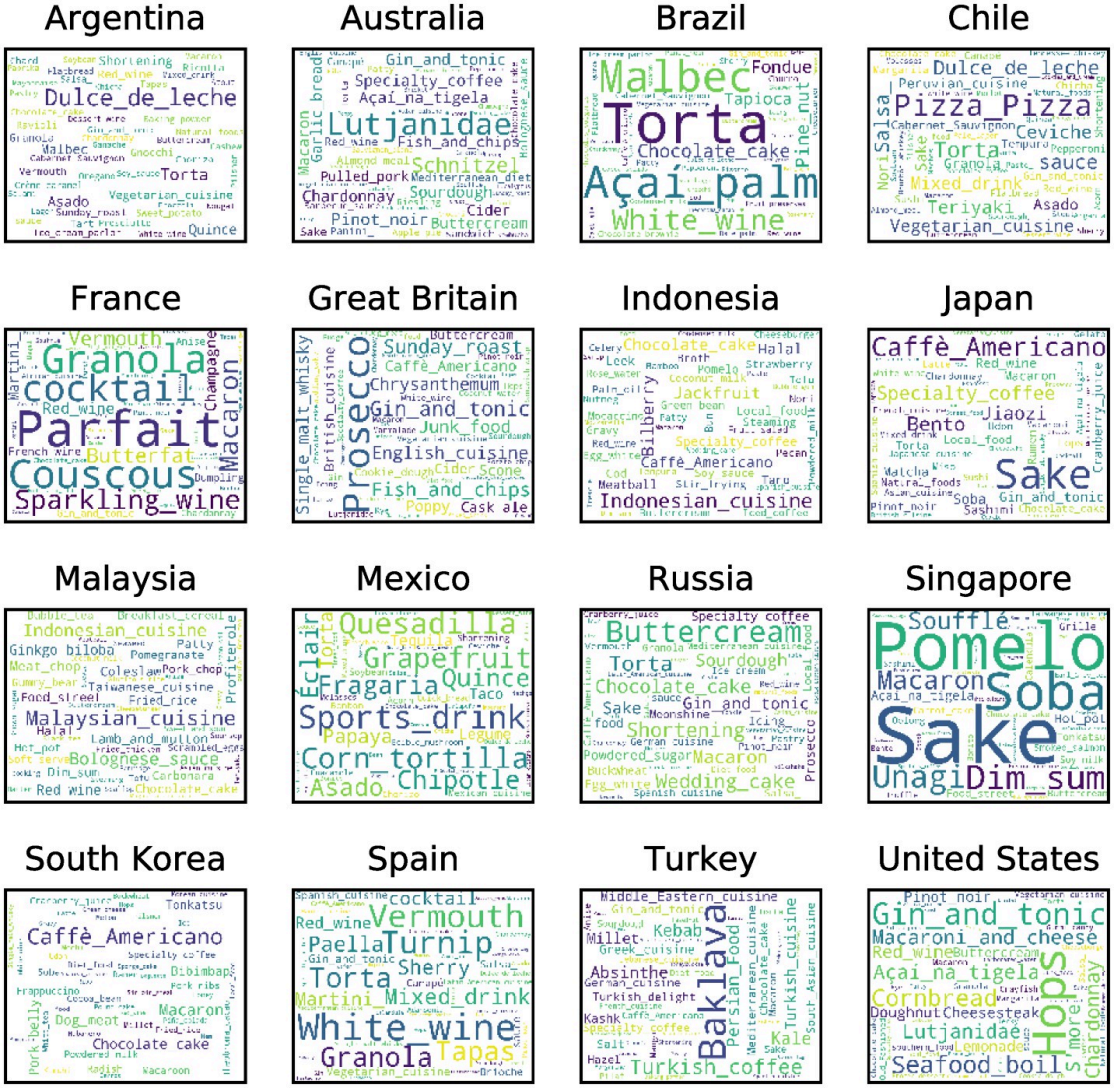
Word clouds showing the names of the 50 food and drink with the largest proportion of the audience in each country, based on data from the Facebook advertisement platform. The size of the words is proportional to the audience interested in the food and drink in the country according to Eq 1. The colors do not have substantive meaning: they are used only to differentiate the words.

Finally, according to Eq 1, for each country, we select the top 50 popular foods and drinks based on the proportion of the Facebook audience interested in each food and drink. For the rest of the paper, we consider only the top foods and drinks selected. Fig 1 shows a word cloud representing the top 50 foods and drinks selected for each country. The words differ in color (colors were chosen randomly) and the size represents the proportion of Facebook users from each country interested in each food and drink according to Eq 1.

#### Vector representation

The Cultural Similarity between countries is measured via a vector, where each position in the vector corresponds to the size of the Facebook audience interested in a food or a drink. Because the audience can also vary greatly across countries, to make a fair comparison between interests in these countries, we need to normalize the audience for each interest by the estimated Facebook population of each country.

We use Eq 2 to normalize the audience for each interest by the Facebook population in each country. Given the number of Facebook users in country *c* (*FB* − *Users*_*c*_), the proportion of the audience *A*_*c*_(*i*) in *c* who is interested in food or drink *i* is given by:

$$\begin{array}{c} A_{c}\left(i\right)=\frac{audience_{c}\left(i\right)}{FB-Users_{c}} \end{array}$$ (2)

**Fig 2:**
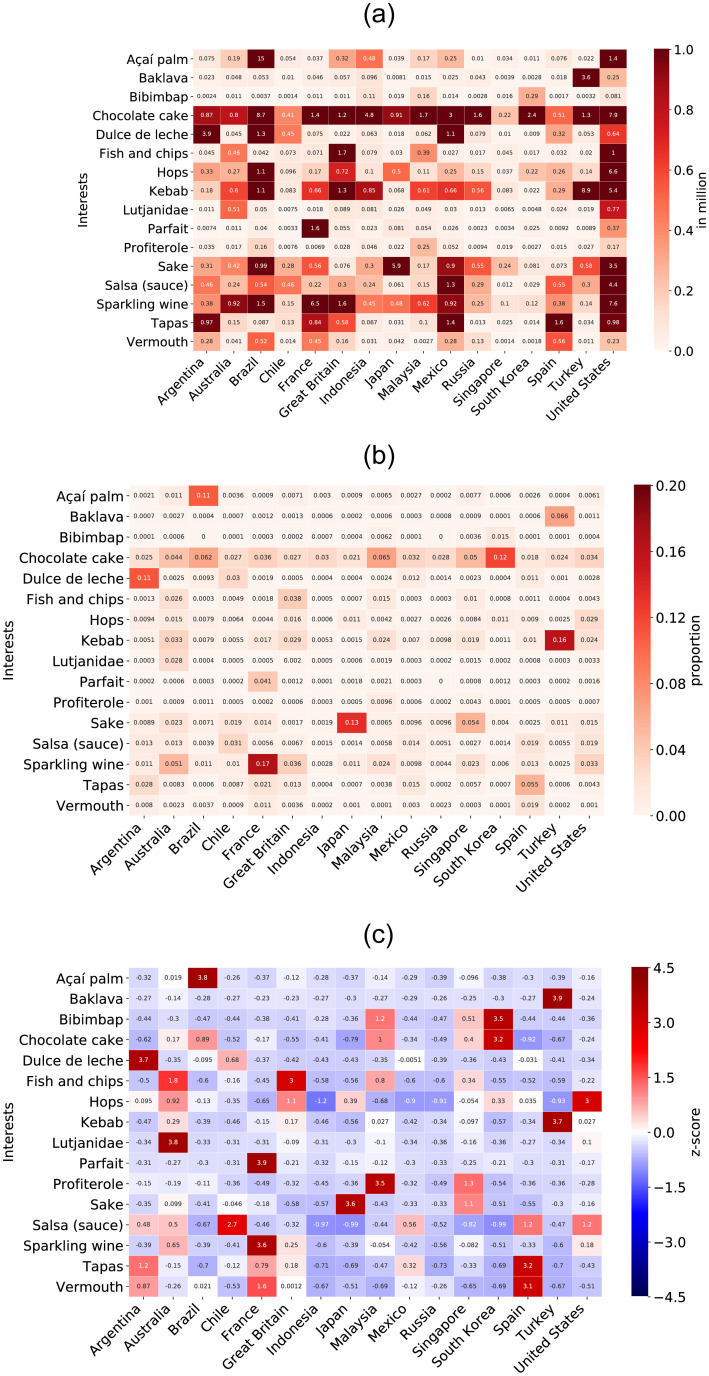
Descriptive statistics on Facebook audience size across countries interested in an illustrative, randomly selected sample of food and drink.

For illustrative purposes, Fig 2a shows the absolute number of Facebook users living in each one of the 16 countries interested in 16 randomly-selected interests. Similarly, Fig 2b shows the same subset of food and drink and the *proportion* of the Facebook audience interested in them for each country. For instance, let us consider the US, which has 230 million Facebook users. Because the number of Facebook users living in the US interested in Kebab is 5.4 million (see Fig 2a), applying Eq 2, 2.4% of the American population on Facebook is interested in Kebab (see Fig 2b). We applied the same operation to all the other foods and drinks.

However, the distribution of foods and drinks is highly unbalanced and some are vastly more popular than others. This difference can impact the similarity measurement between countries because the measure would be disproportionately driven by these few, most popular foods and drinks. For instance, the difference between foods and drinks that have a small proportion of interest in each country will be almost zero, whereas the difference between the most popular food and drink are more likely to be large in relative terms. Note that row *Chocolate cake* and column *United States* in Fig 2a dominate the whole matrix. Observe in Fig 2b that this problem is only partially solved by computing the proportions, as the row *Chocolate cake* continues to be have the highest values for all countries. To give the same importance to all food and drink, we normalize and smooth these distributions by their z-scores as shown in Eq 3.

$$\begin{array}{c} z-score\;\left(A_{c}\left(i\right)\right)=\frac{A_{c}\left(i\right)-E\left[A\left(i\right)\right]}{\sigma (A(i))} \end{array}$$ (3)

where *E*[*A*(*i*)] is the mean of the *A*_*p*_(*i*) across countries, whereas *σ* refers to their standard deviation. In short, the mean is subtracted from the score of each interest and divided by its standard deviation. As a result, each value now represents how many standards deviations an interest in a certain country deviates positively or negatively from the mean. Fig 2c shows the same heatmap as Fig 2b, but considering the z-score normalization. As expected, we observe that the distribution is heterogeneous and does not seem to exhibit a few dominant interests in all the countries. Considering the example in Fig 2c, we can assume that each column represents each country as a vector in terms of their population interests in those foods and drinks.

#### Cultural similarity

The Cultural Similarity (*CS*) between two countries is defined in terms of the Cosine distance between countries with respect to the subset of popular interests in one country. Eq 4 defines this formally: *c*_1_ and *c*_2_ represent two countries, $d_{c_{1}}$ is the subset of interests that are popular in country *c*_1_ that we are considering to generate the vectors $v_{d_{c_{1}}}\left(c_{1}\right)$ and $v_{d_{c_{1}}}\left(c_{2}\right)$. These vectors have elements that represent the level of interest in the country considered for the subset of food and drink that are most popular in country *c*_1_.

$$\begin{array}{c} CS_{A}(c_{1},c_{2}\left|\right|d_{c_{1}})=1-cosine\;dist\left(v_{d_{c_{1}}}\left(c_{1}\right),v_{d_{c_{1}}}\left(c_{2}\right)\right) \end{array}$$ (4)

Note that the measure of cultural similarity between *c*_1_ and *c*_2_ is measured in terms of the *c*_1_ top popular foods and drinks whereas the similarity between *c*_2_ and *c*_1_ is measured in terms of the *c*_2_ top popular foods and drinks. In this case, because the Cultural Similarity between *c*_1_ and *c*_2_ is different from the Cultural Similarity between *c*_2_ and *c*_1_, this measure is not symmetric ($CS_{A}(c_{1},c_{2}\left|\right|d_{c_{1}})\neq CS_{A}(c_{2},c_{1}\left|\right|d_{c_{2}})$). Because of the asymmetry, the measure is named *Asymmetric Cultural Similarity* (*CS*_*A*_). However, we could also measure the similarity between two countries considering a fixed set of interests for both countries. In this case, we can refer to the measure as *Symmetric Cultural Similarity* (*CS*_*S*_) as the Eq 4 can be re-written as shown by Eq 5:

$$\begin{array}{c} CS_{S}(c_{1},c_{2}\left|\right|d^{*})=CS_{B}(c_{1},c_{2}\left|\right|d^{*})=1-cosine\;dist\left(v_{d^{*}}\left(c_{1}\right),v_{d^{*}}\left(c_{2}\right)\right) \end{array}$$ (5)

Eq 5 shows that the Symmetric Cultural Similarity between two countries *c*_1_ and *c*_2_ corresponds to a measure of the Cultural Similarity between them considering all top foods and drinks occurring across all countries, d*. Because the subset of interests is fixed, the Symmetric Cultural Similarity between *c*_1_ and *c*_2_ is equal to the Symmetric Cultural Similarity between *c*_2_ and *c*_1_.

To create a representation via vector with a fixed size for each country while keeping the number of attributes manageable, we consider the top 50 popular foods and drinks for each country. Because the top 50 popular food and drink of several countries overlap because one food or drink can be popular in more than one country, we examined 394 unique interests. In other words, considering the z-scored normalization, we create a representation via vector for each country in terms of their Facebook users’ preferences regarding the 394 most popular foods and drinks selected. Then, for each country, we measure the Symmetric Cultural Similarity by applying the measure given by Eq 5.

## Results

In this section, we present the main results regarding our proposed measures of cultural similarity, the comparison between cultural similarity and the WVS data, and the association between cultural similarity and migration data..

### Patterns of cultural similarity

**Fig 3:**
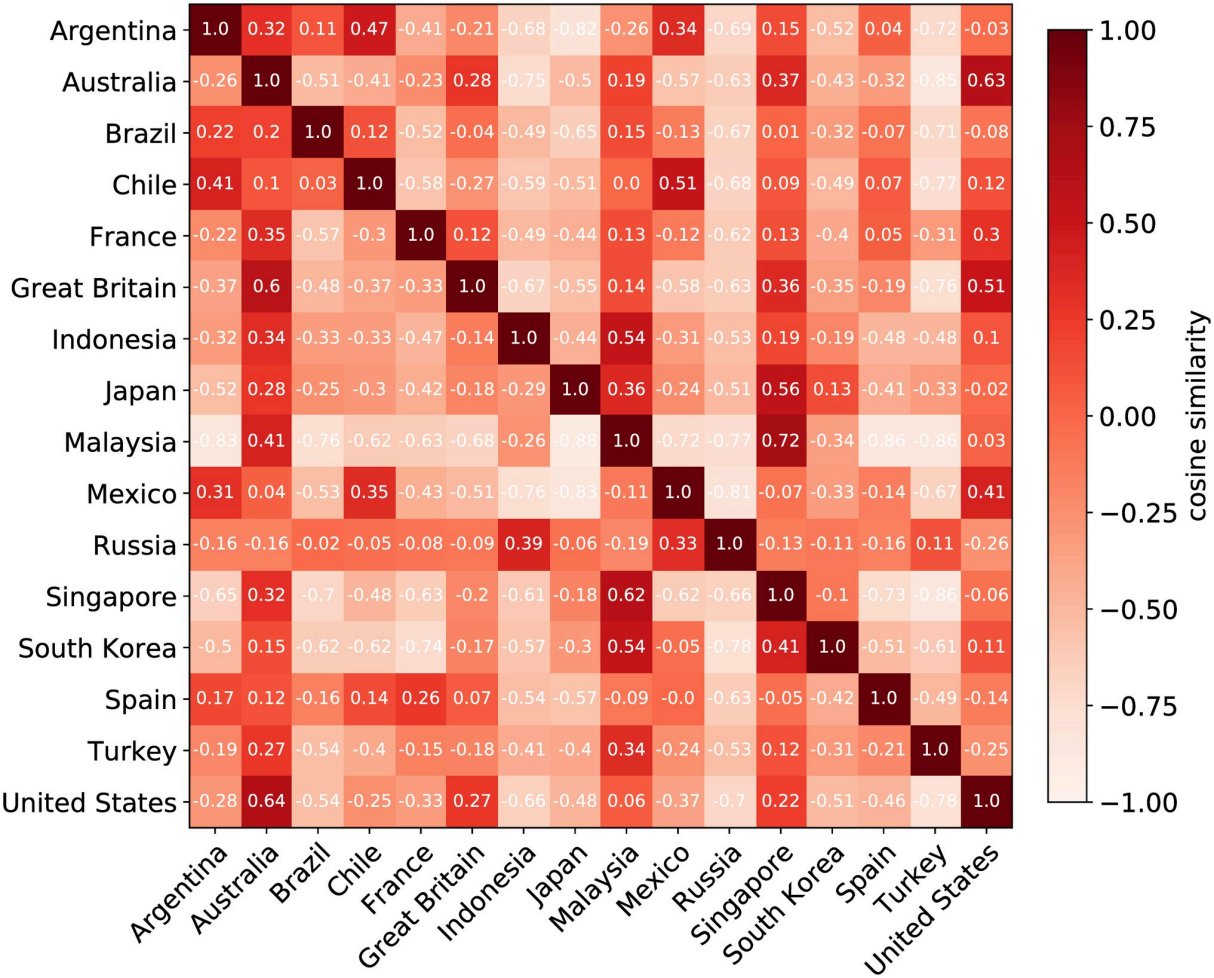
Asymmetric Cultural Similarity. Each cell corresponds to the Cultural Similarity between the country in the row and the country in the column represented via a vector of 50 dimensions considering the top 50 food and drink of the country in the row.

Fig 3 shows the Cultural Similarity between each representation considering the top 50 popular foods and drinks from the country represented by the rows. Notice that because for each row we are considering a different subset of food and drink, the matrix is non-symmetric. As explained before, this measure is therefore named Asymmetric Cultural Similarity.

Notice that the Asymmetric Cultural Similarity shows that some countries are closer to others in terms of their own popular foods and drinks but are more distant from each other when the foreign country’s popular foods and drinks are considered. Some countries such as Indonesia, Japan, Russia, and Turkey are more distant from most of the others in terms of their interest in foreign food and drink. Other countries such as Australia, Great Britain, Malaysia, Singapore, and the United States seem to be more eclectic in their interest in foreign food and drink. Note that Indonesia, Japan, Russia, and Turkey have relatively few immigrants, whereas Australia, Great Britain, and the United States have many. We therefore show that countries with fewer immigrants have more idiosyncratic tastes in food and drink, possibly due to limited exposure to foreign cuisines. Australia, Great Britain, and the United States also appeared the most similar to each others’ food preferences. The exception was the United States which showed that American Facebook users were also highly interested in Mexican food and drink. The similarity between the US and Mexico could be related to the number of Mexican immigrants living there. Since the US is one of the most preferred destinations by immigrants worldwide, we notice that the similarity between the US and the other countries is high when considering interests in foreign food and drink by Facebook users living in the US. However, the opposite is not true—these other countries appeared less interested in American food and drink than the the other way around. Migration can be one reason why some countries’ cultures are more similar to others. However, language and geographic distance also seem to correlate with the cultural similarity. For instance, the English-speaking countries (Australia, Great Britain, and the US), the Spanish-speaking countries (Argentina, Chile, Mexico, and Spain), and the Latin American countries (Argentina, Brazil, Chile) each seemed to be similar to each other. Finally, the matrix in Fig 3 is constructed in a way that countries with high column sums represent more culturally diverse countries with preferences for varied foreign foods, whereas the countries with high row sums represent more culturally influential countries whose food is popular in various other countries.

**Fig 4:**
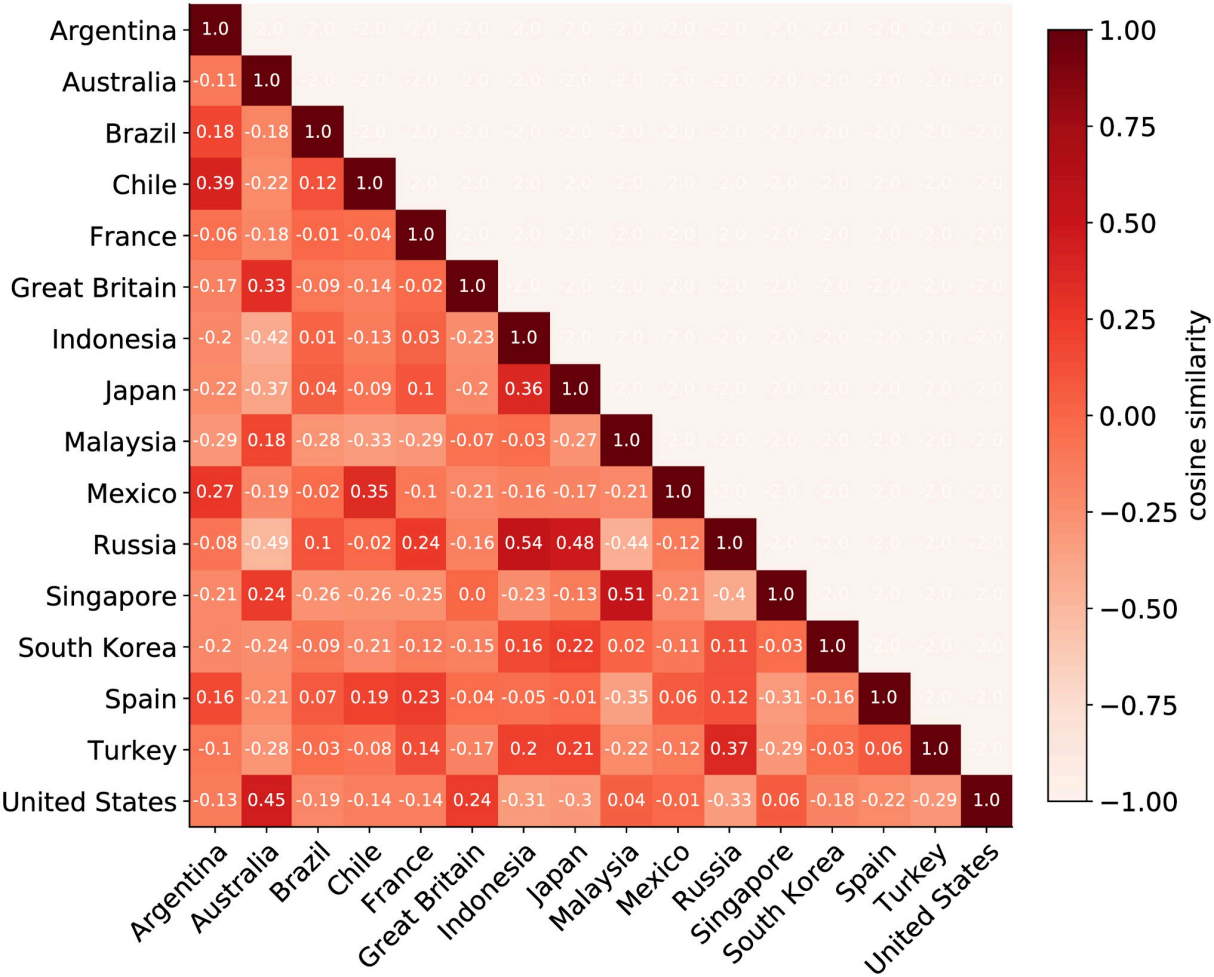
Symmetric Cultural Similarity. Each cell corresponds to the Cultural Similarity between the country in the row and the country in the column represented via a vector of 394 dimensions considering the top 50 food and drink in each country.

Instead of asymmetric measures, we next examine symmetric measures of cultural similarity. Fig 4 shows the Symmetric Cultural Similarity between each pair of countries represented via vector. Note that now the matrix is symmetric and we can name the measure Symmetric Cultural Similarity. In this case we are comparing the countries in terms of their preferences for foods and drinks that are popular around the world. In contrast to the measure of Asymmetric Cultural Similarity, the Symmetric Cultural Similarity provides a broad measure of cultural similarity between countries. For instance, Australia and Indonesia and Australia and Russia are some of the most distant countries from each other. Moreover, Indonesia is the most similar country to Russia. As observed for the Asymmetric Cultural Similarity, language and geographic distance are again associated with low Cultural Similarity because Asian, Latin American, English-speaking, and Spanish-speaking countries, respectively, are culturally close to one another.

### Comparison with the WVS

**Fig 5:**
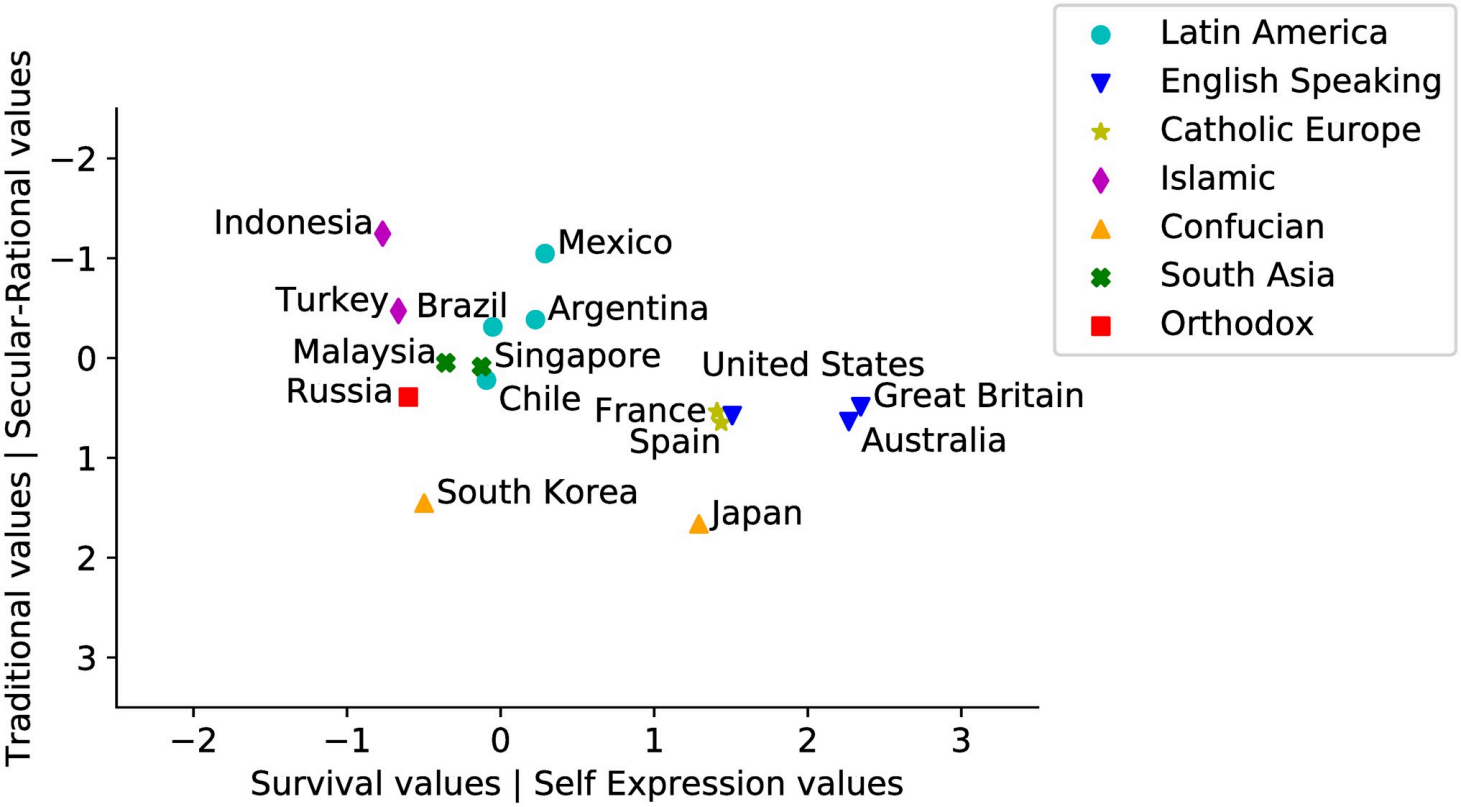
The Inglehart-Welzel world cultural map—WVS 7 (2020).

**Fig 6:**
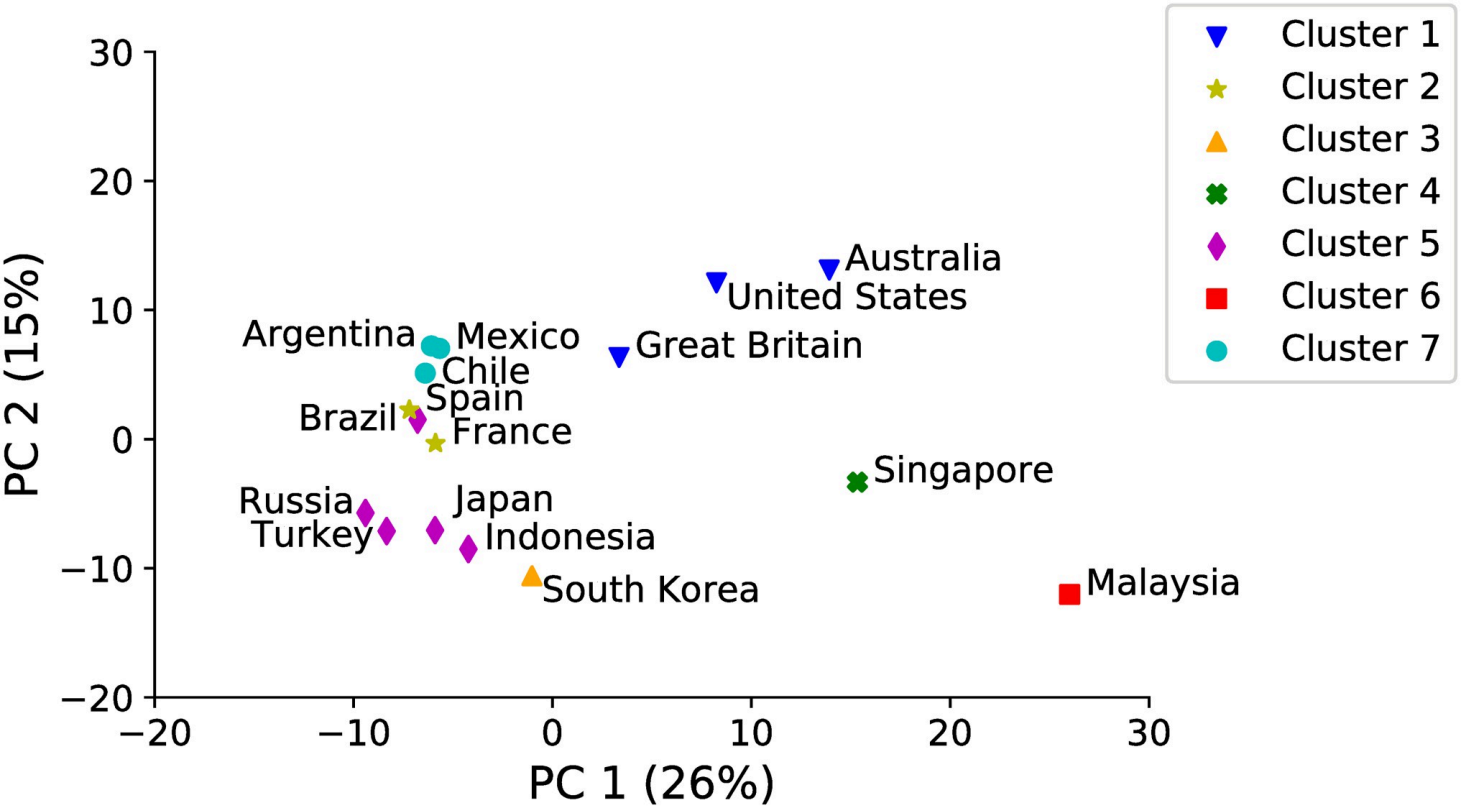
PCA of the Symmetric Cultural Similarity map—Facebook data.

We also contrast our results on Cultural Similarity with other cultural measures based on the World Value Survey (WVS) data. Fig 5 shows the World Value Survey cultural map [10] considering two major dimensions of cross-cultural variation according the WVS data. The map shows each country as part of one of the seven clusters classified by the WVS according to language, religion, or geographic location (Fig 5: Latin America, English Speaking, Catholic Europe, Islamic, Confucian, South Asia, and Orthodox). For simplification, we replicate the WVS cultural map showing only the countries we analyze in this paper. Fig 6 shows the cultural map created by using our Facebook-based cultural vector representation of each country. Because each country is represented by a vector of 394 dimensions, we apply Principal Component Analysis (PCA) over the data to represent the data in two dimensions. The map shows the location of each country in terms of the first and second principal components as well as the variance in the data explained by each one (41% total). Note that we applied the PCA algorithm only to visualize and compare our data the WVS cultural map.

To identify clusters in our Symmetric Cultural Similarity map we use the k-means algorithm, a widely-used clustering technique, to group countries with similar representations over the 394 popular foods and drinks. Each color/symbol represents a cluster obtained by the *k-means* algorithm. We set the parameter *k* = 7 to follow the same number of clusters defined in [10].

Observe that the algorithm correctly identified the English-speaking countries, according to the WVS cultural map, as being part of one cluster (cluster 0) as well as Catholic Europe (France and Spain) as part of another (cluster 1). The Latin American countries (Argentina, Chile, and Mexico) was also in the same cluster (cluster 6), except for Brazil which was included in cluster 4 with Russia, Turkey, Japan, and Indonesia. Finally, South Korea (cluster 2), Singapore (cluster 3), and Malaysia (cluster 5) formed part of different clusters. In general, if we observe the map in Fig 6, the Latin American countries and Catholic Europe were close to each other. However, they were not in the same cluster because we are not identifying the clusters based only on this two-dimensional representation, but based on the 394-dimensional representation of popular foods and drinks.

If we compare the cultural maps in Figs 5 and 6 we observe that the Latin American countries seemed to be more similar to the European countries and Indonesia, Japan, Russia, and Turkey seemed to cluster together in terms of their Facebook users’ interests in popular food and drink. However, we can also observe that Singapore and Malaysia were distant from the other countries, which means that the Facebook users from those countries do not share similar interests in food and drink with other countries.

Finally, by using the PCA of the our Symmetric Cultural Similarity Map, as shown in Fig 6, we formally document the differences between the WVS and our methodology. First, we create the *Symmetric Cultural Similarity ranking* for each target country by sorting the list of countries according to their cosine similarity to the target country. Similarly, the *WVS ranking* for a given country is sorted by the most similar or, in other words, the least distant countries according to their cosine distance in the WVS cultural map. Then, we compute the Jaccard similarity between these two ranks to see if the most similar countries to a specific country are the same with both approaches.

When considering only the top 5 most similar countries to each country, the Jaccard similarity is approximately 0.22. The similarity improves when we consider the top 10 most similar countries (Jaccard similarity = 0.5), which means that our results are relatively close to the WVS in terms of identifying similarity of cultural interests, even though our measure is based only on food and drink popularity.

Similarly, we compared our results with Silva *et al.* [26] to validate our results and ensure comparability with prior research. Similarly, to the *WVS ranking*, we created the *Foursquare ranking* for a given country sorted by the most similar countries according to Silva’s *et al.* cultural map based on food and drink preferences using Foursquare data. The Jaccard similarity between the *Symmetric Cultural Similarity ranking* and the *Foursquare ranking* is approximately 0.44 and 0.59 when we consider, respectively, the top 5 and the top 10 most similar countries to each country. We also compare the Jaccard similarity between the *WVS ranking* and the *Foursquare ranking*. When considering the top 5 and the top 10 most similar countries to each country, the Jaccard similarity is respectively, 0.14 and 0.4. This result indicates that the results obtained from our cultural map improves upon that of prior work in this area. Importantly, these comparisons are limited by the time difference: the data presented by Silva *et al.* are more than 5 years older than our data from Facebook Ads and more than 6 years older than the WVS cultural map. During this time, significant cultural changes may have happened, given that the world is getting more connected every day. Besides the time difference, the Foursquare sample is also expected to be highly unrepresentative because Foursquare is not widely used (e.g., younger people and men are over-represented) and the Foursquare check-ins are limited to people who also have a Twitter account and explicitly post their check-ins, which further skews and reduces the sample. In fact, we expect to see some minor changes between the cultural maps created by using different data sources, however, we decided to keep the comparison with Silva *et al.* to confirm that our methodology produces results in line with other studies.

### Association with migration data

To quantify the association between our measure of Cultural Similarity and migration, we use the migration data provided by the United Nations. By using migration data, we can create, for each country, an *Immigrant ranking* by sorting, in descending order, the countries by the proportion of immigrants from each nationality living in the target country. For instance, the *Immigrant ranking* in Spain would have Great Britain as the origin country of the highest proportion of immigrants living in Spain (immigrants from Great Britain correspond to 0.65% of Spain’s population) followed by Argentina, France, Brazil, Russia, Chile, Mexico, United States, Australia, Japan, South Korea, Turkey, Indonesia, Malaysia, and Singapore. Similarly, we can create a *Cultural Similarity ranking* by sorting the same list of countries according to the Asymmetric Cultural Similarity between countries in terms of the interest in foreign food and drink by Facebook users living in the target country. Notice that, for each country, the *Cultural Similarity ranking* corresponds to its column in Fig 3 sorted in ascending order. Following the same example, the *Cultural Similarity ranking* in Spain would have Chile as the most similar country to Spain in terms of interests in top popular Chilean foods and drinks (the asymmetric cultural distance between Chile and Spain is 0.07) followed by France, Argentina, Brazil, Mexico, Russia, Great Britain, Turkey, Australia, Japan, United States, Indonesia, South Korea, Singapore, and Malaysia.

To understand how strongly the proportion of immigrants living in a country is associated with the cultural similarity between the countries of origin and destination, we measure the correlation between these two rankings. For instance, if we use the Spearman correlation to measure the correlation between the *Immigrant ranking* and the *Cultural Similarity ranking* in Spain, the correlation is equal to 0.82, a high positive correlation. Moreover, we can also compare the rankings using a more intuitive measure by calculating the proportion of countries in the top 5 of the *Cultural Similarity ranking* that are also in the top 5 of the *Immigrant ranking*. In Spain, the top 5 countries in the *Cultural Similarity ranking* are Chile, France, Argentina, Brazil, and Mexico, whereas the top 5 countries in the *Immigrant ranking* are Great Britain, Argentina, France, Brazil, and Russia. Because three (Argentina, France, and Brazil) of the countries in the top 5 of the *Immigrant ranking* are also in the top 5 of the *Cultural Similarity ranking*, it means that 60% of the most similar countries to Spain are also the countries where most immigrants living in Spain come from.

Table 1 shows the Spearman correlation and the *p-value* associated with each correlation for each country. We also present the proportion of countries in the *Cultural Similarity ranking* top 5 that are also present in the *Immigrant ranking* top 5 for each one of the countries. For most of the countries, at least 60% of the *Cultural Similarity ranking* top 5 are also present in the *Immigrant ranking* top 5. Moreover, we observe that for most countries, there is a positive correlation between the number of immigrants from a country and the similarity between Facebook users’ interests in popular food and drink from that country.

**Table 1:** Correlation between the *Immigrant ranking* sorted by the proportion of immigrants living in each country and the *Cultural Similarity ranking* for each country, sorted by the most similar countries in terms of cultural similarity.

Country	Spearman r	Proportion (top 5)
**Spain**	**0.82 (0.0)**	**0.6**
**Malaysia**	**0.75 (0.0)**	**0.6**
**Chile**	**0.74 (0.0)**	**0.6**
**Australia**	**0.65 (0.01)**	**0.8**
**France**	**0.65 (0.01)**	**0.8**
**Great Britain**	**0.62 (0.01**)	**0.8**
**Argentina**	**0.61 (0.02)**	**0.6**
**Mexico**	**0.55 (0.03)**	**0.4**
**Brazil**	**0.54 (0.04)**	**0.8**
**Singapore**	**0.5 (0.06)**	**0.4**
Turkey	0.31 (0.25)	0.4
Russia	0.11 (0.7)	0.6
Japan	0.08 (0.78)	0.2
South Korea	0.07 (0.81)	0.4
United States	0.02 (0.94)	0.4
Indonesia	-0.17 (0.55)	0.2

**Fig 7:**
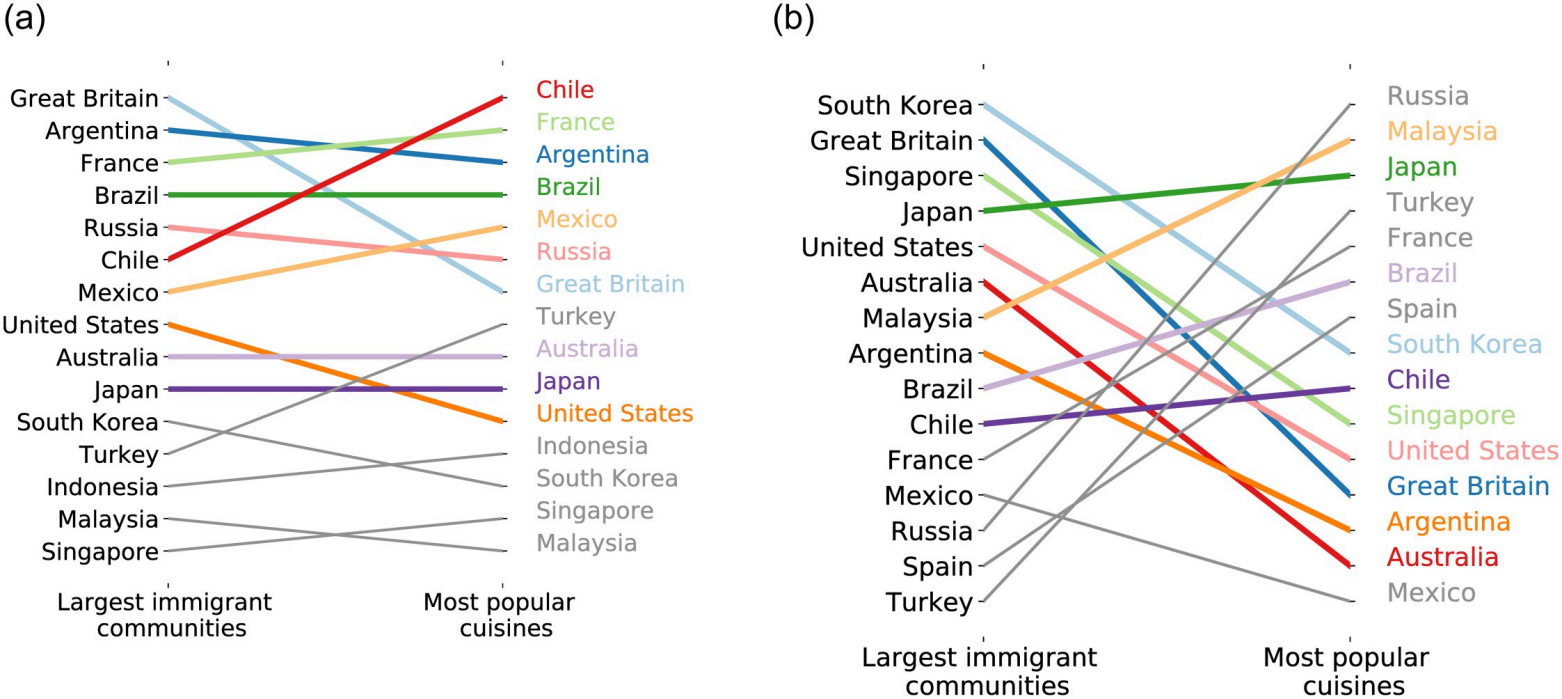
Comparison between the *Immigrant ranking* sorted by the proportion of immigrants living in each country and the *Cultural Similarity ranking* for each country, sorted by the most similar countries in terms of cultural similarity.

The countries shown in Table 1 are sorted according to the Spearman correlation between the *Immigrant ranking* and the *Cultural Similarity ranking*. Spain is the country that shows the highest correlation. Fig 7a illustrates the exact *Immigrant ranking* and the *Cultural Similarity ranking* for Spain in more detail. This pattern illustrates the high positive correlation we found for Spain, which showed that Spain is one of the most preferred destinations by immigrants from Great Britain, Argentina, France, and Brazil, and also shows interest in these countries’ most popular foods and drinks.

However, for other countries, the correlation is lower or even negative and not significantly different from zero given the high p-values. For instance, in Indonesia the number of immigrants is not associated with higher preferences for foreign popular food and drink. Fig 7b illustrates the exact *Immigrant ranking* and the *Cultural Similarity ranking* for Indonesia in more detail. This pattern illustrates the negative correlation we found for Indonesia, which showed that Indonesia is one of the most preferred destinations by immigrants from South Korea, Great Britain, Singapore, and Japan but only Japan’s foods and drinks are popular in Indonesia.

Moreover, there is a positive association between the Spearman correlation of *Immigrant ranking* and the *Cultural Similarity ranking* (Table 1) and the total number of immigrants in the destination country. The Spearman correlation is 0.13 when we consider the total number of immigrants and 0.43 when we sum over the immigrants from the sixteen countries we consider in our work. As outlined above, it is expected that countries with fewer immigrants, such as Indonesia, Russia, Japan, and Turkey, would have more idiosyncratic tastes in food and drink due to limited exposure to foreign cuisines than countries with more immigrants, such as Great Britain and Australia. Finally, it is important to mention that the results are dependent on the subset of countries we are comparing. For instance, not all countries with large immigrant populations in the United States are covered by our data. For example, China is one of the most frequent top origin countries from immigrants in the United States, as well as Indonesia and South Korea [40], but because Facebook is not used in China, we do not have enough information to compare the *Immigrant ranking* and the *Cultural Similarity ranking* considering all possible countries.

## Conclusion

In this work, by using Facebook Ads data, we proposed a scalable approach to obtain proxies for culture in order to measure cultural similarity between countries. Two measures of cultural similarity were proposed, Asymmetric Cultural Similarity and Symmetric Cultural Similarity. Our measures of cultural similarity were compared with the WVS data, and we highlighted some advantages of using social media data in this study. Unlike previous empirical studies, which were based on survey data and large batteries of questions, our methodology is easily scalable, uses passively-collected information internationally available, and considers food and drink as a key dimension which explains a large fraction of the cultural similarities between countries.

Our measure of cultural similarity was also compared with migration data. Our results show that some countries (e.g., Indonesia) are more distant from most of the others in terms of their interest in foreign food and drink. Other countries (e.g., the United States) seem to be more culturally diverse or eclectic in their interest in foreign food and drink, especially if those types of food and drink are popular in countries where most of the immigrants came from. Overall, countries with fewer immigrants have more idiosyncratic tastes in food and drink, possibly due to limited exposure to foreign cuisines. Moreover, in a majority of countries, larger immigrant populations are associated with more similar food and drink preferences between their countries of origin and their destination countries.

Despite the multiple advantages of our proposed methodology using the Facebook Ads data to study cultural similarity, it is important to point out some of the limitations of this approach. First, our analyses are limited to correlational data. Overall, we observed that in a majority of countries, larger immigrant populations are associated with more similar food and drink preferences between their countries of origin and their destination countries. Our hypothesis is that immigrants help bring the culture of their home countries to new countries [41]. More than just a unidirectional process of acculturation, we believe that there is a bidirectional relationship between culture and migration where cultural similarity influences the decision to migrate [2, 42] and the process of migration then leads to increased acculturation and cultural similarity [3, 43, 44]. However, studying the causal pathways between cultural similarity and migration would require longitudinal data instead of a snapshot of a single year. To investigate the causal relationship between cultural similarity and migration longitudinally, we propose to automate the data collection to collect data from Facebook Ads annually. Second, the mechanisms behind the Facebook Advertising Platform are a black box and some information, such as interests, can be inferred by Facebook based on unknown algorithms. Previous research showed that demographic information, such as sex, location, and age, is accurately reported by Facebook users or estimated by Facebook [38]. As Facebook’s business model relies on targeted advertisements related to interests, we expect that Facebook’s models identify users’ interest in a fairly accurate way. However, it would be important for future research to independently validate whether interests, too, are accurately captured by the Facebook Advertising Platform. Third, our conclusions are limited to the sample of countries selected. The cultural similarities between countries, as well as the correlation between cultural similarity and migration, may differ depending on the subset of countries chosen, which is also constrained by our data source (e.g., Facebook is not used in China). However, our measure of asymmetric cultural similarity between any two countries in our sample would be unaffected if we included other countries because this measure is defined solely on the basis of top dishes in these two countries. When it comes to other metrics and other parts of the world, future research should investigate the extent to which our conclusions can be generalized by replicating our approach for more countries, as well as by considering additional metrics. An exciting area of further development includes incorporating culture and our methods within the perspective of social network analysis. Within this context, shared interests in food and drinks can be seen as a defining component of relationships between countries. This approach would build and enrich the growing literature on identifying the role and boundaries of migration networks [45–48] and the relationship between cultural production and migration [49]. Finally, as a matter of fact, it is important to emphasize that while the cuisine of a country is an important cultural marker to study cultural similarity, the proposed methodology could be extended other types of attributes and interests, which might be relevant for studies with other goals. In this sense, future research can shed new light on the importance of additional cultural markers to characterize and measure cultural similarity between countries, as well as the network structure of relationships between countries.

The methodology that we proposed in this article is based on a scalable approach that makes use of social media data to study the cultural similarity between countries. The proposed measure of cultural similarity, even if it is just focusing on food and drink as cultural markers, seems to capture a substantial portion of the variability measured in data from the WVS. In addition to that, our measure of cultural similarity takes into consideration factors such as international data availability, time, cost, and asymmetry, which have hampered previous efforts to study cultural similarity. Finally, the high correlation between cultural similarity between countries and the number immigrants in the country suggests that cultural similarity related to food and drink preferences can be considered among relevant predictors of migration, in addition to more established quantities which include economic variables, trade [50] or indicators of cultural diffusion in networks, such as communication and the flow of information [51].
